# Characterisation of Post-Sepsis Cardiomyopathy Using Cardiovascular Magnetic Resonance

**DOI:** 10.3390/diagnostics15080997

**Published:** 2025-04-14

**Authors:** Samuel Malomo, Thomas Oswald, Edward Stephenson, Anthony Yip, Thomas Alway, Stanislav Hadjivassilev, Steven Coombs, Susan Ellery, Joon Lee, Rachael James, Claire Phillips, Barbara Philips, David Hildick-Smith, Victoria Parish, Alexander Liu

**Affiliations:** 1 Sussex Cardiac Centre, Royal Sussex County Hospital, Brighton BN2 5BE, UK; s.malomo@nhs.net (S.M.); thomas.oswald@nhs.net (T.O.); e.stephenson4@nhs.net (E.S.); anthony.yip2@nhs.net (A.Y.); t.alway@nhs.net (T.A.); stanislav.hadjivassilev@nhs.net (S.H.); steven.coombs@nhs.net (S.C.); susan.ellery1@nhs.net (S.E.); joon.lee@nhs.net (J.L.); rachael.james3@nhs.net (R.J.); david.hildick-smith@nhs.net (D.H.-S.); victoria.parish@nhs.net (V.P.); 2 Intensive Care Unit, Royal Sussex County Hospital, Brighton BN2 5BE, UK; claire.phillips20@nhs.net (C.P.); barbara.philips@nhs.net (B.P.); 3 Brighton and Sussex Medical School, Brighton BN1 9PX, UK

**Keywords:** sepsis, cardiomyopathy, cardiovascular magnetic resonance, heart failure, cardiovascular imaging

## Abstract

**Background:** Post-sepsis cardiomyopathy is associated with an increased risk of adverse cardiovascular outcomes. It remains poorly understood, which limits therapeutic development. This study characterised post-sepsis cardiomyopathy using cardiovascular magnetic resonance (CMR) imaging. **Methods:** Patients admitted with acute sepsis and suspected cardiac injury or heart failure who subsequently (47 days [IQR: 22–122]) underwent CMR at a UK tertiary cardiac centre were included. Age- and gender-matched controls (n = 16) were also included. Subjects underwent CMR at 1.5 Tesla with cines, native T1- and T2-mapping, and late gadolinium enhancement (LGE) imaging. **Results:** Of the 22 post-sepsis patients (age 50 ± 13 years; 64% males), 13 patients (59%) had left ventricular (LV) dilatation. Patients had significantly elevated left ventricular (LV) end-diastolic and end-systolic volume indices compared to controls (*p* = 0.011 and *p* = 0.013, respectively). Eleven patients (50%) had LV systolic dysfunction (ejection fraction < 50%), most of whom (8/11) had non-ischaemic patterns of LGE (n = 7 mid-wall; n = 1 mid-wall/patchy). In the eleven patients with preserved LV systolic function (ejection fraction ≥ 50%), three patients (27%) had significant LGE (n = 1 mid-wall; n = 1 subepicardial/mid-wall; n = 1 patchy). Compared to controls, patients had elevated septal native myocardial T1 values (*p* < 0.001) but similar septal native myocardial T2 values (*p* = 0.090), suggesting the presence of myocardial fibrosis without significant oedema. **Conclusions:** Post-sepsis cardiomyopathy is characterised by LV dilatation, systolic dysfunction, and myocardial fibrosis in a non-ischaemic distribution. Significant myocardial oedema is not prominent several weeks post-recovery. Further work is needed to test these findings on a multi-centre basis and to develop novel therapies for post-sepsis cardiomyopathy.

## 1. Introduction

Sepsis is a leading cause of morbidity and mortality worldwide, contributing to over 11 million deaths each year [1]. Approximately a quarter of acutely septic patients develop cardiac dysfunction, which can persist after recovery from sepsis and is associated with an adverse clinical prognosis [2,3,4,5]. Patients with acute sepsis can develop cardiac dysfunction as a result of the acute proinflammatory response and the vascular mal-adaptations of sepsis, such as peripheral vasodilatation and increased peripheral vascular permeability [6]. This acute sepsis-related cardiomyopathy is thought to be reversible within a couple of weeks [6,7,8]. However, increasing evidence suggests that a proportion of sepsis survivors develop cardiac dysfunction, which may be a different disease entity to the cardiomyopathy which occurs during acute sepsis [8]. The cardiac phenotype and pathophysiology of this population of patients with post-sepsis cardiomyopathy (cardiac structural or functional abnormalities in patients who have recovered from acute sepsis) remain poorly understood, which limits the development of novel therapeutic targets.

Survivors of sepsis have up to a two-fold increased risk of developing major adverse cardiovascular outcomes, which cannot be wholly explained by their pre-sepsis risk factor profiles and the acute septic event [9]. Recent evidence suggests that acutely septic patients develop myocardial oedema and stress-related cardiomyopathic changes [10,11]. It remains unclear whether these changes persist after recovery from acute sepsis or whether myocardial oedema translates to the development of myocardial fibrosis in the chronic stages of sepsis. Further, the prevalence of cardiac dysfunction in post-sepsis patients and the myocardial tissue characteristics underpinning cardiac dysfunction are poorly understood, which hinders the effective clinical management of this patient group. Advancing our understanding of post-sepsis cardiomyopathy is an important clinical priority.

Cardiovascular magnetic resonance (CMR) imaging provides multi-parametric and non-invasive assessment of cardiac structure, function, and tissue characterization [12,13]. CMR is considered a reference standard method for the evaluation of cardiac biventricular volumes and systolic function [12]. Late gadolinium enhancement (LGE) imaging also provides a detailed assessment of myocardial infarction and the delineation of non-ischaemic patterns of focal myocardial fibrosis [12]. Over the last two decades, parametric mapping methods, such as native T1- and T2-mapping, have added to the diagnostic value of CMR for cardiac involvement in inflammatory conditions and the detection of diffuse myocardial fibrosis and myocardial oedema [13,14].

This study sought to characterise the cardiac structure, function, and myocardial tissue in sepsis survivors with suspected cardiac injury or heart failure, as compared to controls. We hypothesised that sepsis survivors have reduced cardiac systolic function and increased myocardial fibrosis.

## 2. Methods

### 2.1. Study Subjects

Patients over the age of 18 years admitted to the University Hospitals Sussex National Health Service (NHS) Foundation Trust (UK) between March 2014 to October 2024 with acute sepsis and suspected myocardial injury or heart failure, who subsequently underwent clinical cardiovascular magnetic resonance (CMR) were included in the study. CMR scans were performed at the Royal Sussex County Hospital, Brighton (a UK tertiary cardiac centre). Patients were excluded if their CMR scans were non-diagnostic (n = 1) or if the date of onset of sepsis was unclear (n = 2). A total of twenty-two patients were included in the study.

To provide a comparison to patients and to calibrate for site- and scanner-specific native T1 and T2 values by parametric mapping methods, 16 controls without a recent history of sepsis were also included. Controls were overall age- and gender-matched to the post-sepsis patients and consisted of subjects referred for clinical CMR who were subsequently found to have normal biventricular volumes systolic function with no significant myocardial LGE, as adjudicated by the clinical CMR consultant.

### 2.2. Ethical Approval Statement

This retrospective study was approved by the Research and Innovation Department of the University Hospitals Sussex NHS Foundation Trust, and informed patient consent was waived.

### 2.3. Clinical Data Collection

Clinical parameters of the patients and controls were collected from the electronic medical records. These included demographic data, cardiac symptoms, co-morbidities, and regular medications. For patients, details regarding the acute septic episode were also collected. The data were independently validated by a second observer as referenced to the electronic medical records.

### 2.4. Cardiovascular Magnetic Resonance (CMR)

Patients and controls underwent CMR at 1.5 Tesla with cines, native parametric mapping (T1- and T2-mapping where possible), and LGE imaging, as previously described [12,13]. Twenty-one subjects (n = 13 patients; n = 8 controls) underwent scans with a Siemens scanner (Aera, Siemens Healthineers, Erlangen, Germany). In August 2023, a new MRI centre was opened in our hospital, and the remaining study subjects (n = 9 patients; n = 8 controls) were scanned using a Philips scanner (Ingenia Ambition, Philips Healthcare, Best, The Netherlands).

Cine imaging was performed in long- and short-axis views [12]. Native T1-mapping was performed in a mid-ventricular short-axis slice using vendor-provided Modified Look-Locker Inversion recovery (MOLLI) sequences with a 5s(3s)3s scheme on both Siemens and Philips scanners. Native T2-maps were also acquired using vendor-provided sequences (T2Map True FISP for Siemens; GraSE 9 echo sequence for Philips) in the same slice positions as T1-maps. LGE imaging was performed in matching long- and short-axis views to cines, approximately 8 min after an intravenous bolus of gadolinium-based contrast agent (0.1 mmol/kg; Dotarem, Guerbet, France), followed by a 15 mL saline flush.

### 2.5. CMR Image Analysis

Cardiac volumes and systolic function were analysed using commercially available software (cvi42, Circle Cardiovascular Imaging, Calgary, AB, Canada) by experienced clinical CMR consultants. LGE images were assessed visually by the same CMR consultants, noting the location and extent of enhancement. Native T1- and T2-maps were analysed without reference to other clinical data by an experienced observer using the Sectra Uni-view platform. Septal native myocardial T1 and T2 values were estimated by the manual placement of regions of interest (ROI) in the septum, carefully avoiding areas with artefacts, areas of LGE, and the partial volume of blood–myocardium interface. Global native myocardial T1 and T2 values were estimated by the manual placement of ROI in a near-closed “C” shape to include as much of the global LV myocardium as possible, also carefully avoiding areas with artefacts and the partial volume of blood–myocardium interface. The T1- and T2-map ROI and data were independently verified by a second observer.

### 2.6. Statistical Analysis

**Figure 1:**
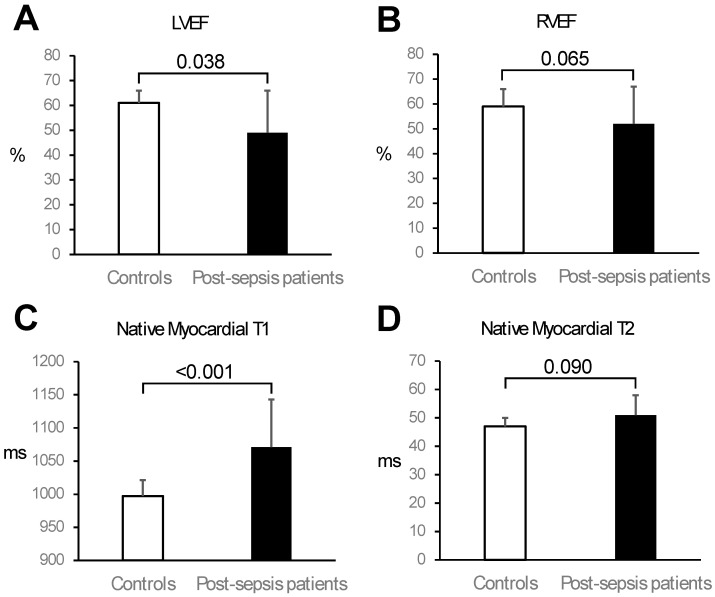
Comparison of cardiac function and tissue characterisation parameters between controls and post-sepsis patients. Compared to controls, patients had significantly lower left ventricular (LV) ejection fraction (EF) (Panel (**A**)) and similar right ventricular (RV) EF (Panel (**B**)). Moreover, compared to controls, patients had significantly higher mean septal native myocardial T1 values (Panel (**C**)) and similar mean native myocardial T2 values (Panel (**D**)). Bars represent mean, and error bars represent one standard deviation.

Data were checked for normality using the Kolmogorov–Smirnov test. Parametric data were presented as mean ± standard deviation (SD). Non-parametric data were presented as median [interquartile range; IQR]. LVEFs for patients were presented as mean ± SD despite having a non-parametric distribution to enable conventional error bar display in the bar chart in Figure 1. Parametric continuous data were compared using the independent sample *t*-test. Non-parametric data were compared using the Mann–Whitney test. Categorical data were compared using Fisher’s exact test. *p* values < 0.05 denotes statistical significance. Data were analysed using commercially available software (MedCalc, version 20.104, Mariakerke, Belgium). All data analyses and final results were independently validated by two additional observers.

## 3. Results

### 3.1. Acute Sepsis Events in Patients

Of the 22 patients admitted for acute sepsis (50 ± 13 years; 64% males; median hospital stay: 15 days [IQR 11–33]), pneumonia was the commonest cause (64%), followed by sepsis of unknown origin (14%), abscess (9%), gastrointestinal (9%), and cellulitis (5%; Table 1). Thirteen patients (59%) required intensive care unit (ICU) admission; three patients (14%) required high-dependency unit (HDU) admissions; two patients (9%) required management in a cardiac care unit, and four patients (18%) received treatment on the medical wards (Table 1). Four patients (18%) required intubation and mechanical ventilation, six patients (27%) required vasopressor support, and six patients required inotropic support (27%; Table 1).

During the acute sepsis episode, patients had elevated C-reactive protein levels (CRP; peak 270 ± 134 mg/L) and white cell count (peak 18.5 × 10^9^/L [IQR: 14.5–26.4]). The peak high-sensitivity cardiac troponin T (hs-cTnT) levels were also elevated (108 ng/L [15–841]; Table 1). Thirteen patients (59%) had transthoracic echocardiographic (TTE) evidence of severe LV systolic dysfunction (LVEF < 35%) during their acute sepsis episode. One patient (5%) had TTE evidence of regional wall motion abnormalities. Three patients (14%) had pericardial effusions, and one patient (5%) had significant LV hypertrophy. Table 1 summarises the clinical details of patients during their acute sepsis episode.

### 3.2. Demographics and Clinical Data of Post-Sepsis Patients and Controls

Patients and controls had similar age (50 ± 13 years vs. 43 ± 14 years; *p* = 0.128), gender profiles (64% male vs. 71% male; *p* = 1.000), body mass index (26 ± 6 kg/m^2^ vs. 24 ± 3 kg/m^2^; *p* = 0.349) and body surface area (1.9 ± 0.3 m^2^ vs 1.9 ± 0.2 m^2^ *p* = 0.793; Table 2). Patients and controls also had a similar prevalence of cardiac symptoms, including chest pain (*p* = 0.450), palpitations (*p* = 0.098), dyspnoea (*p* = 0.165), pre-syncope or syncope (*p* = 0.065; Table 2).

No patient had a pre-existing history of heart failure or chronic kidney disease (Table 2). Patients and controls had similar levels of co-morbidities including atrial fibrillation (*p* = 0.203) hypertension (*p* = 0.374), diabetes mellitus (*p* = 0.624), smoking (ex- or current; *p* = 0.370), hypercholesterolaemia (*p* = 1.000), ischaemic heart disease (*p* = 0.499), COPD/asthma (*p* = 1.000) and cerebrovascular accident/transient ischaemic attack (*p* = 1.000; Table 2).

Medical therapies, including sacubitril/valsartan, mineralocorticoid receptor antagonists, sodium–glucose co-transporter-2 inhibitors, loop diuretics, and anticoagulation, were more prevalent in patients compared to controls. Other cardiac medications were similar between the two groups (Table 2).

### 3.3. Cardiac Volumes and Function in Patients and Controls

In patients, CMR was performed at a median of 47 days [IQR 22–122] after acute sepsis (Table 3). Thirteen patients (59%) had left ventricular (LV) dilatation. Compared to controls, patients had significantly higher LV end-diastolic volume index (98 ± 33 mL/m^2^ vs. 77 ± 13 mL/m^2^; *p* = 0.011) and LV end-systolic volume index (45 mL/m^2^ [28–58] vs. 30 ± 7 mL/m^2^; *p* = 0.013 by Mann–Whitney test; Table 3).

Patients had significantly lower LV ejection fraction (LVEF) compared to controls (49 ± 17% vs. 61 ± 5%; *p* = 0.038; Table 3 and Figure 1). Eleven patients (50%) had LV systolic dysfunction (LVEF < 50%), which was severe (LVEF < 35%) in four patients (18%). Patients also had significantly greater LV mass index than controls (75 ± 18 g/m^2^ vs. 55 ± 13 g/m^2^; *p* < 0.001; Table 3). Right ventricular (RV) volumes and systolic function were similar between patients and controls as assessed by CMR (Table 3 and Figure 1).

**Figure 2:**
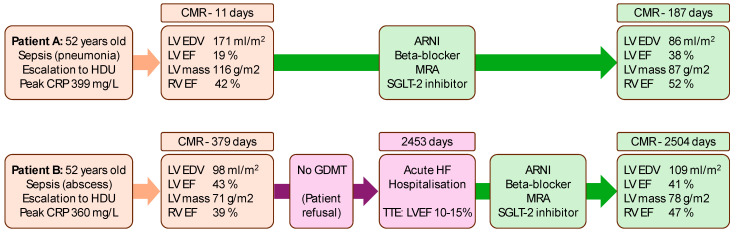
Serial cardiovascular magnetic resonance (CMR) assessments in two post-sepsis patients in relation to medical therapy. Patient A had early CMR assessment post-sepsis, which showed severely increased left ventricular (LV) end-diastolic volumes (EDV) with severely reduced LV ejection fraction (EF). Several months after guideline-directed medical therapy (GDMT) for heart failure, LV EDV was reduced, and LVEF improved. Patient B had deferred CMR assessment post-sepsis, which uncovered non-dilated LV with moderately reduced LVEF. GDMT was not commenced owing to patient refusal to take medications. The patient was hospitalised a few years later with acute heart failure decompensation with transthoracic echocardiogram (TTE) demonstrating severe LV systolic dysfunction. GDMT was commenced, and the LV function stabilised on repeat CMR within 2 months. ARNI: angiotensin receptor-neprilysin inhibitor; CRP: C-reactive protein; HDU: high dependency unit; MRA: mineralocorticoid receptor antagonist; RV: right ventricular.

Two illustrative examples of post-sepsis patients and their clinical journeys are shown in Figure 2.

### 3.4. Myocardial Tissue Characterisation

**Figure 3:**
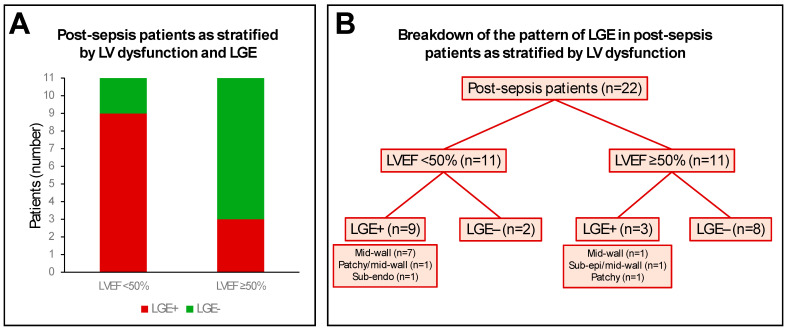
Pattern of late gadolinium enhancement (LGE) in post-sepsis patients. Panel (**A**) shows the relative distribution of LGE positive (LGE+) and LGE negative (LGE−) post-sepsis patients as stratified by left ventricular (LV) systolic dysfunction, using an LV ejection fraction (LVEF) cut-off of 50%. Panel (**B**) shows a breakdown of the LGE patterns in post-sepsis patients as stratified by LVEF cutoff of 50%. Sub-endo: subendocardial; sub-epi: subepicardial.

Focal myocardial fibrosis, as detected by LGE, was present in 12 patients (55%). Mid-wall LGE was present in 10 patients; patchy LGE in 2 patients; subepicardial LGE in 1 patient; and subendocardial LGE in 1 patient (Table 3). In the 11 patients with LV dysfunction (LVEF < 50%), 9 patients (82%) had significant LGE (n = 7 mid-wall; n = 1 patchy/mid-wall; n = 1 subendocardial; Figure 3). In the other 11 patients (preserved LV function; LVEF ≥ 50%), 3 patients (27%) had significant LGE (n = 1 mid-wall; n = 1 subepicardial/mid-wall; n = 1 patchy; Figure 3). No controls had any discernible LGE. No RV LGE was observed in patients or controls.

Owing to two different CMR scanners being used in the study, native myocardial T1 and T2 values were compared between the two scanners for calibration purposes (Table 4). Controls scanned on Siemens and Philips scanners (n = 8 each) had similar native myocardial T1 values in the septum (*p* = 0.576) and globally (*p* = 0.881), as well as similar native myocardial T2 values in the septum (*p* = 0.496) and globally (*p* = 0.267; Table 4).

Native myocardial T1 mapping was performed in 20/22 patients, and native myocardial T2 mapping was performed in 12/22 patients. Both T1 and T2 mapping were performed in all 16 controls. Compared to controls, post-sepsis patients had significantly higher native myocardial T1 values in the septum (1071 ± 72 ms vs. 997 ± 24 ms; *p* < 0.001) and globally (1064 ± 69 ms vs. 996 ± 28 ms; *p* < 0.001; Table 3 and Figure 1). Patients and controls had similar native myocardial T2 values in the septum (51 ± 7 ms vs. 47 ± 3 ms; *p* = 0.090) and globally (51 ± 6 ms vs. 47 ± 3 ms; *p* = 0.063; Table 3 and Figure 1).

### 3.5. Clinical CMR Diagnoses

**Figure 4:**
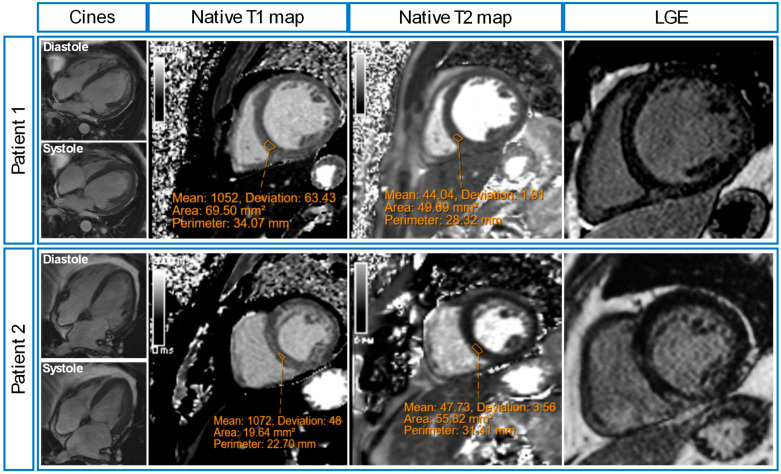
Illustrative cardiovascular magnetic resonance (CMR) images of two post-sepsis patients. Patient 1 was admitted to high high-dependency unit with severe pneumonia, and a transthoracic echocardiogram (TTE) showed severe LV systolic dysfunction. His post-sepsis CMR scan showed left ventricular (LV) dilatation with severe systolic dysfunction (LVEF 19%), elevated native myocardial T1 values (1052 ± 63 ms), normal native myocardial T2 values (44 ± 2 ms) and mid-wall distribution of late gadolinium enhancement (LGE). Patient 2 was admitted to the intensive care unit with severe pneumonia, and a TTE showed severe LV systolic dysfunction. His post-sepsis CMR showed LV dilatation with mild systolic dysfunction (LVEF 49%), elevated native myocardial T1 values (1072 ± 48 ms), normal native myocardial T2 values (48 ± 4 ms), and mid-wall distribution of LGE.

Of the post-sepsis patients, 13 patients (59%) had appearances of non-ischaemic cardiomyopathy (n = 8 dilated left ventricle; n = 3 likely previous myocarditis; n = 1 inflammatory cardiomyopathy; n = 1 stress-induced cardiomyopathy). Of the remaining nine patients, one patient had myocardial infarction, two patients had pericardial effusions, and six patients had unremarkable CMR scans (Table 3). Figure 4 shows illustrative CMR images of two post-sepsis patients with non-ischaemic cardiomyopathy appearances.

## 4. Discussion

This study is the first to provide a multi-parametric characterisation of sepsis survivors using CMR, as compared to controls. The main findings are that in sepsis survivors: (i) cardiac systolic dysfunction is prevalent (50% of patients had LVEF < 50%); (ii) focal LV myocardial fibrosis is prevalent (55% of patients had LGE presence); (iii) diffuse LV myocardial fibrosis is likely present (elevated septal and global native myocardial T1 values); (iv) myocardial oedema may not be a prominent feature (similar native myocardial T1 values to controls); and (v) many post-sepsis patients have features resembling non-ischaemic cardiomyopathies, previous myocarditis, inflammatory or stress induced cardiomyopathies. These findings should be validated further on a multi-centre basis, which can potentially facilitate the future development of therapies for post-sepsis cardiomyopathy.

### 4.1. The Post-Sepsis Cardiomyopathy Phenotype

The aetiology of cardiac dysfunction in acute sepsis is multi-factorial, involving deranged cardiac loading conditions, increased systemic vascular permeability, and peripheral vasodilatation [7]. The proinflammatory cytokine storm can also exert adverse effects on myocardial contractility [7]. Although cardiac dysfunction seen in acute sepsis is often considered a reversible phenomenon [2,15,16], the current study suggests that such cardiac dysfunction can persist in many patients after recovery from sepsis, as assessed by CMR.

In patients with persisting LV systolic dysfunction post-sepsis, the data in this study showed that many cases demonstrated LV dilatation with focal myocardial fibrosis as detected by LGE. Moreover, the myocardial fibrosis was predominantly mid-wall in distribution, resembling features of non-ischaemic cardiomyopathies [17]. LV dilatation was apparent in over half of the study patients despite the instigation of guideline-directed heart failure medical therapy post-sepsis [18]. One explanation may be that the CMR scans were performed relatively early (median 47 days post-sepsis) since it can take several months for LV dilatation to regress on therapy [18]. Since myocardial fibrosis can develop in the early stages of non-ischaemic cardiomyopathy [19], the presence of LGE in post-sepsis patients may suggest early myocardial remodelling. Further studies are required to better understand the temporal relationships between myocardial fibrosis development and LV structural remodelling in sepsis survivors. Serial cardiac imaging studies are also required to better understand the degree and natural history of myocardial fibrosis progression in post-sepsis patients. Existing studies on the use of LGE to characterise LV functional recovery in patients post coronary revascularisation [20] and LV functional recovery after medical and/or cardiac device therapy [21] demonstrate the ability of the heart to reverse remodeling. The effect of cardiac therapies in patients with post-sepsis cardiomyopathy would also benefit from further investigation in these avenues.

Although some cases of post-sepsis patients were given a likely diagnosis of previous myocarditis, there was a paucity of subepicardial LGE distribution. This suggests that the myocardial inflammation/fibrosis may be different from that encountered in the cases of viral myocarditis with subepicardial involvement. This notion deserves further investigation. Only one case of myocardial infarction was found in the post-sepsis patients, which was correlated to the finding of significant coronary artery disease. Although this patient suffered sepsis, the cause of the cardiomyopathy was more likely to be due to ischaemic heart disease. Whilst the study showed a number of non-ischaemic patterns of myocardial fibrosis in post-sepsis cardiomyopathy, these may be distinguishable from other patterns in conditions such as cardiac amyloidosis and sarcoidosis [22]. There is currently a paucity of data comparing post-sepsis cardiomyopathy and other forms of inflammatory heart diseases, such as cardiac sarcoidosis [21]. Further work is needed to compare these phenotypes of cardiomyopathies.

### 4.2. Diffuse Myocardial Fibrosis Post-Sepsis

Autopsy studies in septic patients demonstrated evidence of inflammatory cell infiltration, such as by neutrophils, into the myocardial interstitial space [23,24,25], leading to extra-cellular fibrotic expansions and diffuse myocardial fibrosis [7,15,26,27,28]. In this study, the observation of increased native myocardial T1 values in post-sepsis patients, remote to focal LGE, may be explained by the presence of diffuse fibrosis [13]. However, owing to the lack of CMR scans for pre-sepsis, it remains unclear whether any diffuse myocardial fibrosis detected was pre-existing or formed *de novo* as a result of the acute sepsis event. Although post-contrast T1-maps were acquired in the scanning protocol, haematocrit was not routinely taken on the day of the clinical CMR study owing to logistical reasons, which prohibited the calculation of extra-cellular volume (ECV) fractions; this biomarker should be further studied.

Currently, there are no long-term follow-up data on post-sepsis cardiomyopathy patients, which would provide greater insights into the natural history of this condition. The lack of studies on the therapeutic strategies for cardiomyopathy in sepsis survivors also limits the clinical confidence with which clinicians can treat these patients. Further work is needed to better understand the clinical prognosis of patients with phenotypical evidence of post-sepsis cardiomyopathy and to assess the efficacy of existing and novel therapies.

### 4.3. Myocardial Oedema Post-Sepsis

Patients with acute sepsis demonstrate evidence of myocardial oedema as indicated by increased T2-weighted signals on CMR [10,11]. We had expected to observe myocardial oedema on the post-sepsis CMR scans since oedema signals are known to persist up to a few months after the acute inflammatory insult [29]. However, the lack of elevated native myocardial T2 values, as compared to controls, suggested that the acute septic inflammation may have settled. Studies with serial follow-up cardiac imaging are required to further test this hypothesis. Further, factors such as sepsis severity, the timing of the CMR studies, and the sensitivity of different T2-mapping sequences should also be tested in future studies to better understand the evolution of myocardial oedema post-sepsis.

The lack of CMR scanning during the index septic event meant that we could not study the degree of myocardial oedema in the acute period as a comparator for the subsequent CMR scan. Not all patients underwent T2-weighted imaging, such as short tau inversion recovery (STIR), which may have shed further light on the presence/absence of myocardial oedema [13]. Despite these factors, the absence of significant myocardial oedema by native T2-mapping in many post-sepsis patients means that anti-inflammatory therapies may not be comprehensive treatment options for post-sepsis cardiomyopathy. This idea is consistent with observations that anti-inflammatory therapies have had mixed effectiveness in patients with severe sepsis [30,31,32]. Potential therapies which target adverse cardiac remodelling, rather than focusing wholly on kerbing the systemic inflammation, may be a viable option for further clinical therapeutic development.

### 4.4. Limitations and Future Directions

The retrospective nature and the relatively small sample size meant that the results were prone to sampling bias. The results should be validated in a larger study conducted on a multi-centre basis with serial CMR studies. The data from a larger validation study would answer a number of further questions, such as (i) the effect of sepsis severity on the development of post-sepsis cardiomyopathy, (ii) the effect of pre-existing co-morbidities of patients on the severity and characteristics of post-sepsis cardiomyopathy, (iii) the effect of demographics data such as gender, race, and socio-economic class on the susceptibility of patients to develop post-sepsis cardiomyopathy. No patient underwent endomyocardial biopsy (EMB), which would have enabled the correlation between CMR parameters and histological findings. However, EMB is not commonly performed in septic patients and tends to be performed to rule out other serious diagnoses, such as fulminant myocarditis. Further, the study findings suggested that myocardial fibrosis can be mid-wall and patchy in post-sepsis patients, meaning that EMB may be prone to sampling errors. The lack of serum biomarkers, such as interleukins and tumour necrosis factor, meant that the precise inflammatory process during sepsis and the trajectory of inflammatory recovery post-sepsis could not be correlated to the CMR findings. However, the highly elevated CRP, WCC, and cardiac troponins during sepsis indicated that both significant inflammatory response and myocardial injury took place. Although the study patients did not have a pre-existing history of heart failure prior to their sepsis event, the lack of pre-sepsis CMR meant that it was difficult to ascertain whether the myocardial fibrosis found in the study was wholly due to the sepsis event. Future larger population-based studies with pre-sepsis CMR scans would help to better answer this question. Owing to the sample size, meaningful relationships could not be assessed with the patients’ clinical outcomes, which deserves further investigation.

## 5. Conclusions

Post-sepsis cardiomyopathy is characterised by LV dilatation, systolic dysfunction, and myocardial fibrosis in a non-ischaemic distribution. Significant myocardial oedema is not prominent several weeks post-recovery. Further work is needed to test these findings on a multi-centre basis and to develop novel therapies for post-sepsis cardiomyopathy.

## Figures and Tables

**Table 1:** Clinical features of the sepsis event in patients.

	Patients (n = 22)
Age, years	50 ± 13
Male	14 (64)
Sepsis cause	
Pneumonia	14 (64)
Unknown origin	3 (14)
Abscess	2 (9)
Gastrointestinal	2 (9)
Cellulitis	1 (5)
Duration of hospital stay, days	15 [11–33]
Care escalation	
ICU	13 (59)
HDU	3 (14)
CCU	2 (9)
Ward-based care	4 (18)
Support requirements	
Intubation	4 (18)
Vasopressor	6 (27)
Inotrope	6 (27)
Serum biomarkers	
Peak CRP, mg/L	270 ± 134 (n = 20)
Peak WCC, ×10^9^/L	18.5 [14.5–26.4] (n = 20)
Peak Hs-cTnT, ng/L	108 [15–841] (n = 15)
Echocardiography findings	
Severe LV dysfunction	13 (59)
RWMA	1 (5)
Pericardial effusion	3 (14)
LVH	1 (5)

CCU: cardiac care unit; CRP: C-reactive protein; HDU: high dependency unit; Hs-cTnT: high sensitivity cardiac troponin T; ICU: intensive care unit; LV: left ventricular; LVH: LV hypertrophy; RWMA: regional wall motion abnormality; and WCC: white cell count. Continuous variables are displayed as mean ± SD or median [interquartile range]. Categorical variables were displayed as number (%).

**Table 2:** Baseline clinical characteristics of post-sepsis patients and controls.

	Patients (n = 22)	Controls (n = 16)	*p* Value
Age, years	50 ± 13	43 ± 14	0.128
Male	14 (64)	10 (71)	1.000
BMI, kg/m^2^	26 ± 6	24 ± 3	0.349
BSA, m^2^	1.9 ± 0.3	1.9 ± 0.2	0.793
Cardiac symptoms			
Chest pain	4 (18)	5 (31)	0.450
Palpitations	5 (23)	8 (50)	0.098
Dyspnoea	10 (45)	3 (19)	0.165
Pre-syncope/Syncope	1 (5)	5 (31)	0.065
Co-morbidities			
Atrial fibrillation	6 (27)	1 (6)	0.203
Hypertension	4 (18)	1 (6)	0.374
Diabetes mellitus	3 (14)	1 (6)	0.624
Smoking (ex- or current)	5 (23)	1 (6)	0.370
Hypercholesterolaemia	3 (14)	2 (13)	1.000
CKD	0 (0)	0 (0)	-
Heart failure	0 (0)	0 (0)	-
Ischaemic heart disease	2 (9)	0 (0)	0.499
COPD/Asthma	4 (18)	2 (13)	1.000
CVA/TIA	1 (5)	0 (0)	1.000
Medications			
Anti-platelet drugs	6 (27)	1 (6)	0.203
Beta-blocker	13 (59)	7 (44)	0.512
ACE-inhibitor/ARB	5 (23)	1 (6)	0.370
Sacubitril/Valsartan	6 (27)	0 (0)	0.030
MRA	11 (50)	0 (0)	<0.001
SGLT-2 inhibitor	8 (36)	0 (0)	0.012
Digoxin	1 (5)	0 (0)	1.000
Loop diuretics	7 (32)	0 (0)	0.014
Statin	6 (27)	4 (25)	1.000
Anticoagulation	6 (27)	0 (0)	0.030

ACE: angiotensin-converting enzyme; ARB: angiotensin receptor blocker; BMI: body mass index; BSA: body surface area; CKD: chronic kidney disease; COPD: chronic obstructive airways disease; CVA: cerebrovascular accident; MRA: mineralocorticoid receptor antagonist; SGLT-2: Sodium-glucose co-transporter-2; and TIA: transient ischaemic attack. Continuous variables are displayed as mean ± SD or median [interquartile range]. Categorical variables were displayed as number (%).

**Table 3:** Cardiac imaging data of post-sepsis patients and controls.

	Patients (n = 22)	Controls (n = 16)	*p* Value
Age, years	50 ± 13	43 ± 14	0.128
Male	14 (64)	10 (71)	1.000
Day from sepsis to CMR	47 [22–122]	-	-
CMR volumes and function			
LV EDVi, mL/m^2^	98 ± 33	77 ± 13	0.011
Dilated LV	13 (59)	-	-
RWMA	4 (18)	-	-
LV ESVi, mL/m^2^	45 [28–58]	30 ± 7	0.013
LV SVi, mL/m^2^	47 ± 16	48 ± 8	0.897
LV EF, %	49 ± 17	61 ± 5	0.038
LV EF <50%	11 (50)	-	-
LV EF <35%	4 (18)	-	-
RV EDVi, mL/m^2^	82 ± 23	80 ± 19	0.774
RV ESVi, mL/m^2^	39 [26–47]	33 ± 11	0.267
RV SVi, mL/m^2^	42 ± 14	46 ± 9	0.262
RV EF, %	52 ± 15	59 ± 6	0.065
LV mass index, g/m^2^	75 ± 18	55 ± 13	<0.001
Dilated LA	11 (50)	5 (31)	0.326
Dilated RA	7 (32)	3 (19)	0.469
LGE data			
LV LGE present	12 (55)	-	-
Mid-wall	10 (45)	-	-
Subepicardial	1 (5)	-	-
Patchy	2 (9)	-	-
Subendocardial	1 (5)	-	-
LV LGE location			
Lateral	6 (27)	-	-
Septal	5 (23)	-	-
Anterior	3 (14)	-	-
Inferior	1 (5)	-	-
RV LGE present	0 (0)	-	-
Native myocardial T1			
Septal, ms	1071 ± 72 (n = 20)	997 ± 24	<0.001
Global, ms	1064 ± 69 (n = 20)	996 ± 28	<0.001
Native myocardial T2			
Septal, ms	51 ± 7 (n = 12)	47 ± 3	0.090
Global, ms	51 ± 6 (n = 12)	47 ± 3	0.063
Probably diagnosis on CMR			
Dilated left ventricle	8 (36)	-	-
Myocarditis	3 (14)	-	-
Inflammatory cardiomyopathy	1 (5)	-	-
SICM	1 (5)	-	-
Myocardial infarction	1 (7)	-	-
Pericardial effusion	2 (9)	-	-
Unremarkable scan	6 (27)	16 (100)	-

CMR: cardiovascular magnetic resonance; EDVi: end-diastolic volume index; EF: ejection fraction; ESVi: end-systolic volume index; FDG: LA: left atrium; LGE: late gadolinium enhancement; LV: left ventricular; RA: right atrium; RV: right ventricular; RWMA: regional wall motion abnormality at rest; SICM: stress-induced cardiomyopathy; SVi: stroke volume index; T1: spin-lattice relaxation time; T2: spin–spin relaxation time. Continuous variables are displayed as mean ± SD or median [interquartile range]. Categorical variables were displayed as number (%).

**Table 4:** Scanner-specific native myocardial T1 and T2 values of controls.

	Philips (n = 8)	Siemens (n = 8)	*p* Value
Native myocardial T1			
Septal, ms	993 ± 17	1000 ± 30	0.576
Global, ms	995 ± 25	997 ± 33	0.881
Native myocardial T2			
Septal, ms	48 ± 3	47 ± 2	0.496
Global, ms	48 ± 4	46 ± 2	0.267

T1: spin–lattice relaxation time; T2: spin–spin relaxation time. Continuous variables are displayed as mean ± SD.

## Data Availability

Patient clinical data in the study cannot be publicly shared, but anonymised version can be provided on reasonable request to the corresponding author.
